# Renewing *PLOS Medicine*’s editorial board in times of global distraction

**DOI:** 10.1371/journal.pmed.1002239

**Published:** 2017-02-28

**Authors:** The PLOS Medicine Editors

**Affiliations:** Public Library of Science, San Francisco, California, United States of America and Cambridge, United Kingdom

## Abstract

At a time when medical research must compete increasingly with spin, bias, and competing interests, the *PLOS Medicine* Editors see a crucial role for our renewed Editorial Board.

The vitality and validity of medical research cannot be taken for granted. In the United States alone, recent months have seen an industry effort to silence researchers who identify dangerous ingredients in dietary supplements, and scientifically baseless calls to revisit the evidence underlying infant vaccination [1,2]. Particularly troubling is the possibility that health may have incubated the kind of thinking that has come to define a “post-truth era” in politics and the media: “In many ways, the health world is one in which facts don’t matter, experts are suspect, and relativistic approaches to testable phenomena are widely accepted” [3].

A defining role for science is to persist when less objective aspects of human experience threaten to eclipse well-founded knowledge. Perhaps the adage (attributed to Abraham Lincoln) that “you cannot fool all the people all the time” must ever coexist with the roughly contemporary observation (perhaps by P. T. Barnum) that “no one ever lost a dollar underestimating the taste of the American public.” There is little reason to believe that either saying applies uniquely to the US. In a world at risk of distraction from major ongoing health challenges, including population growth, migration, emerging infectious diseases, aging populations, and unaffordability of care—to name just a few—medical journals must regularly rededicate our efforts to uphold scientific integrity.

While editorial staff bear responsibility for processes to promote and maintain research quality, it is editorial board members who establish a journal’s legitimacy within the scientific community that the journal exists to serve. Staff editors rely on editorial board members to guide our topical priorities at a time of rapid change in the conditions that affect medical research and health itself. We depend on our editorial board members as partners in improving the peer review and transparent reporting processes that set verifiable science apart from the welter of unfiltered (or disingenuously selected) assertions that vie for readers’ attention. We need their involvement, alongside that of peer reviewers, to ensure that the journal reflects the research community’s highest values of quality, ethics, and relevance to human health.

In October 2016, the *PLOS Medicine* staff editors surveyed our editorial board members—who serve both as academic editors for submitted manuscripts and as advisors on editorial policy—about the journal’s topical priorities. The most frequent response was strong support for the journal’s ongoing relevance to global health, particularly as outlined in the United Nations Sustainable Development Goals. Other high priorities included research of clear relevance to clinical care and to health policy (including disease prevention, antimicrobial resistance, noncommunicable diseases, and environmental effects on health) and encouragement of innovative and interdisciplinary research methods. Research on health systems and implementation science, translational research that bridges human biology and clinical application, analysis of large-scale datasets, and research integrity (including the effects of corporate influence) all received multiple mentions. We believe these updated priorities build on *PLOS Medicine’s* existing areas of strength and will stand the journal in good stead during times of rapid, unpredictable change.

We also asked the editorial board members whether they would favor closer collaboration in shaping the journal’s direction. We proposed more regular interactions with staff editors, not only in evaluating submitted manuscripts, but also in determining what research we should seek to publish and how best to engage on an ongoing basis with research communities to ensure that the journal’s scope reflects their evolution. The majority of responding board members supported this idea. Predictably, many longstanding board members have attained greater responsibility in their institutions and communities than at the time they were first invited to serve, but several of these more senior members recommended “rising star” colleagues. In the ensuing weeks, the staff editors reached out to many of these and to others known to us from their exemplary contributions as authors, peer reviewers, or guest academic editors.

Having now completed this initial phase of the editorial board renewal process, we are indebted to past board members who have supported the journal with wise counsel, often over many years. We are grateful to continuing board members who enthusiastically agreed to remain in a more active capacity. And, as will be clear from our current editorial board roster (http://journals.plos.org/plosmedicine/s/editorial-board), we have every reason to be gratified and excited by the talent and energy that our newly invited members generously offer. Our collective goal is to ensure that *PLOS Medicine* remains a journal of and by the medical research community and for a wider readership who value access, relevance to health, and research quality.

There can be no place in science for a “post-truth era.” Even at a time when health research and evidence-based policy must contend with an onslaught of spin, bias, and competing interests on a daily basis, we believe research communities that engage fully with their journals can maintain the integrity of medical research. We look forward to working closely with our renewed Editorial Board.
